# Evaluation of single-nucleotide polymorphism imputation using random forests

**DOI:** 10.1186/1753-6561-3-s7-s65

**Published:** 2009-12-15

**Authors:** Daniel F Schwarz, Silke Szymczak, Andreas Ziegler, Inke R König

**Affiliations:** 1 Institut für Medizinische Biometrie und Statistik, Universität zu Lübeck, Universitätsklinikum Scleswig-Holstein, Campus Lübeck, Maria-Goeppert-Str. 1, 23562 Lübeck, Germany

## Abstract

Genome-wide association studies (GWAS) have helped to reveal genetic mechanisms of complex diseases. Although commonly used genotyping technology enables us to determine up to a million single-nucleotide polymorphisms (SNPs), causative variants are typically not genotyped directly. A favored approach to increase the power of genome-wide association studies is to impute the untyped SNPs using more complete genotype data of a reference population.

Random forests (RF) provides an internal method for replacing missing genotypes. A forest of classification trees is used to determine similarities of probands regarding their genotypes. These proximities are then used to impute genotypes of untyped SNPs.

We evaluated this approach using genotype data of the Framingham Heart Study provided as Problem 2 for Genetic Analysis Workshop 16 and the Caucasian HapMap samples as reference population. Our results indicate that RFs are faster but less accurate than alternative approaches for imputing untyped SNPs.

## Background

Recently, genome-wide association studies (GWAS) have expanded our knowledge about genomic variants that influence susceptibility to complex diseases such as myocardial infarction [1,2]. One important reason for this success is the substantial technological progress enabling the genotyping of up to a million single-nucleotide polymorphisms (SNPs) simultaneously. However, with about 15 million known SNPs in the current Build 129 of dbSNP http://www.ncbi.nlm.nih.gov/SNP and almost four million of these available in release 23a from the HapMap project http://www.hapmap.org, the coverage achieved by direct genotyping is still far from perfect. Thus, the majority of all known SNPs in the genome are evaluated only indirectly with commonly used genotyping platforms. Consequently, today's GWAS are usually not able to genotype causal variants but will detect association with a nearby SNP in high linkage disequilibrium (LD). Although this approach has proved successful in many cases, it is still likely that a great number of causal variations are yet undetected and that the power of GWAS could be increased by performing statistical tests with disease influencing SNPs directly [3].

One preferred approach to increase the power of GWAS is to combine data from several studies [4], thus increasing the sample sizes from thousands to tens of thousands. However, these meta-analyses of GWAS pose special problems, such as a limited overlap in genotyped SNPs if different platforms were used across the studies. A promising solution is to impute the respective untyped SNPs using genotype data of the performed study and data of a similar reference population that has been genotyped at additional SNPs [5]. As a result, estimated genotypes may be used to fill in the gaps in the original GWAS and to increase the overlap between different GWAS.

In our work, we applied the random forests (RF) imputation approach to untyped SNPs [6]. We evaluate this method on genotype data of probands of the Framingham Heart Study provided as Problem 2 for Genetic Analysis Workshop (GAW) 16.

## Methods

### Algorithm

RF is a data-mining method that is able to produce accurate classifiers even when many variables are observed in relatively few individuals. Furthermore, it provides estimates of variable importance, generates an unbiased generalization error estimate, and includes a technique for estimating missing data. Using its classifying power, we have recently shown that a screening of SNPs by RF is suitable to detect promising candidate SNPs in GWAS for complex diseases [7].

A specific feature of RF is the ability to replace missing values through an iterative process [8]. To use this for imputation, two essential prerequisites need to be satisfied. First, each variable must have at least one non-missing value. This is not fulfilled in presence of untyped SNPs because all values are missing. Adding non-missing data of a reference population is a possible approach to overcome this as described in Algorithm 1 Step 1a. Second, RF needs a variable to classify on because RF is a supervised learning method. If the data contain only genotypes, this precondition is not met. Algorithm 1 Step 1b describes a solution to use genotype data by enriching it with synthetic data.

**Figure 1:**
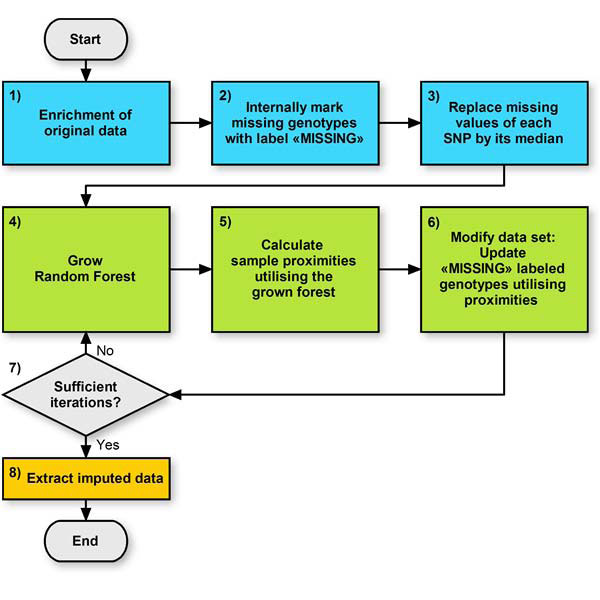
**Flow chart of algorithm 1**. Algorithm 1 proceeds as follows: 1) enrich data; 2) mark missing and undefined genotypes; 3) roughly replace missing values; 4) grow forest; 5) calculate sample proximities; 6) update former missing values using proximities; 7) repeat Steps 4-6 several times; 8) extract imputed original data.

Thus, the original method was modified accordingly. The procedure for imputing missing SNPs comprises several steps as shown in Figure 1 and proceeds as follows:

### Algorithm 1

#### 1) Enrichment of original data

The data is enriched in two successive steps as follows:

a) The original data is merged with a subset of HapMap [9] genotype data. This subset contains exactly the SNPs that were typed in the original data. If one also aims at imputing SNPs that were not typed at all in the original data, these SNPs need to be contained in the HapMap subset as well. The selected HapMap probands have to be independent from each other and chosen from a population similar to the probands in the original data.

b) The data is subsequently modified as follows: to begin, the original data set that contains only genotype information is considered as Class 1. A new synthetic data set with the same number of probands and SNPs is created and labelled as Class 2. This synthetic data is created by sampling at random without replacement from the univariate distributions of the original data. The sampling is separately performed for each SNP. Thus, each SNP has the same univariate distribution as the corresponding SNP in the original data. The random SNPs of Class 2 are independently distributed and contain no dependency structure [8]. The original data and the synthetic data are merged into a single data set, resulting in an artificial two-class data set that can be used by supervised learning methods. The RF is thus able to perform unsupervised learning so that phenotype data is not mandatory [8,10].

#### 2) Labelling

Missing and undefined genotypes are internally marked with the label ***MISSING***.

#### 3) Rough imputation

Missing values of each SNP are roughly imputed by its median value. This initial crude imputation is essential because RF cannot handle missing data [8].

#### 4) Forest growing

A classification forest is grown. The trees are built on the new data set created in Step 1.

#### 5) Calculate probands' proximities

An important part of the RF imputation method is the proximity matrix, which contains the pair-wise similarities between all pairs of probands. Specifically, the proximities are determined as follows: first, they are set to zero. Then, each proband is classified by all trees in the forest. For each tree, if two probands are evaluated by exactly the same series of decision rules, their proximity is increased by one. A detailed description of decision tree rules and classification is given in Breiman et al. [11].

#### 6) Updating *MISSING *genotypes

Genotypes internally marked as ***MISSING ***are re-estimated. The updating is separately performed for each SNP. The new value is calculated as follows: at a specific SNP, each proband holds exactly one genotype value of the set 0, 1, or 2. This also applies to former missing genotypes that were roughly imputed or updated during a previous iteration. For each proband with a genotype marked as ***MISSING***, the new genotype is calculated using a weighted average of the genotypes of remaining probands. Each weight is calculated based on the proximity between the two samples as determined in Step 5.

#### 7) Iterate

It is recommended to perform Steps 4-6 at most five times [8].

#### 8) Imputed data

The resulting data set of this iterative procedure consists of HapMap data, imputed original data, and synthetic data. The imputed original data set is extracted and the imputation is finished.

Algorithm 1 was implemented in C++ language.

### Evaluation of imputation

The assessment of the imputation quality was performed as follows:

1) A subset of the Framingham Heart Study [12] genotype data containing SNP genotypes of independent probands was chosen.

2) From all available SNPs in the original data set, 10% were drawn without replacement. All genotypes of these SNPs were deleted in this data set to mimic a situation with untyped SNPs.

3) Imputation of the deleted SNPs was performed as described in Algorithm 1.

4) For each SNP, the imputed genotypes were compared with the corresponding original genotypes. The imputation accuracy of a SNP equals the number of correctly imputed genotypes divided by the number of all imputed genotypes. The result reflects the quality of the SNP's imputation.

Quality and computing time of the imputation depend primarily on two parameters, namely, the number of trees in each forest and the number of iterations in Algorithm 1. To obtain the optimal trade-off between computing time and imputation quality, several RF imputation runs were performed using different parameter settings. In addition, a potential correlation of SNP imputation quality with minor allele frequency (MAF) was subsequently investigated.

As a standard approach, the untyped SNPs were also imputed using the computer program IMPUTE [5] using default parameters and option pgs. IMPUTE calculates three probabilities for each SNP genotype of a sample. Each probability belongs to the homozygote rare allele, homozygote common allele, or heterozygote genotype. The most likely genotype has been chosen for missing replacement. Results of IMPUTE and our RF method were subsequently compared with regard to accuracy and computing time.

### Data

Data from 6752 participants in the Framingham Heart Study [12] were provided as Problem 2 for GAW16. For our analysis, only the genotypes of 762 unrelated individuals from generation 3 were selected; neither haplotype data nor LD block data were used. Standard quality control was applied to genotype data of the Affymetrix GeneChip^® ^Human Mapping 500k Array Set (488,146 SNPs) as recommended [1,2,4,13]. SNPs with a call rate < 0.98 per study group, a MAF < 0.01 in the cases and controls combined or a *p*-value < 0.0001 for deviation from Hardy-Weinberg equilibrium in control group were excluded, resulting in 336,206 SNPs. Finally, only SNPs of chromosome 22 were selected. The resulting data contained the genotypes of 3,775 SNPs. The mean distance between two adjacent SNPs amounted to 3488 bp. About 10% (*n *= 376) of the SNPs were deleted to represent untyped SNPs in a real-world data set as previously described. The mean MAF of SNPs was 0.2286, with a standard deviation of 0.1348. The minimal MAF was 0.0134 and MAF maximum was 0.4993. The mean distance between an untyped SNP and the nearest genotyped SNP amounted to 3916 bp. For reference HapMap data [9] used in Algorithm 1 Step 1a, genotypes of the 3,775 SNPs in 60 unrelated CEU founders were downloaded.

## Results

**Figure 2:**
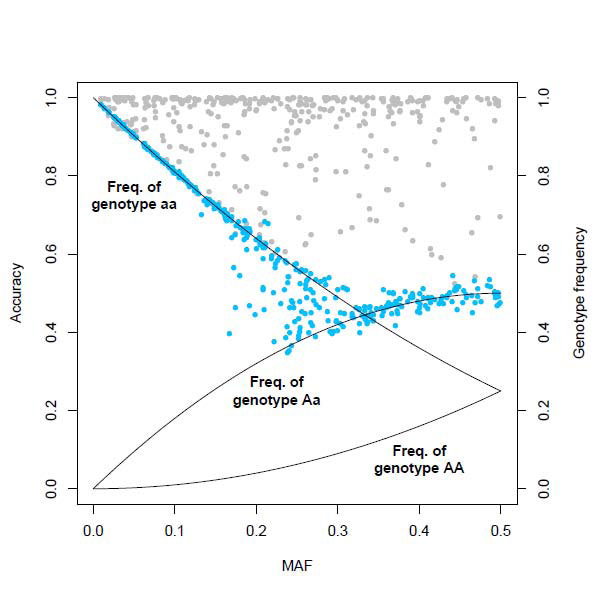
**Correlation between imputation accuracy and MAF**. Blue and gray dots denote 3,775 SNPs that were imputed by RF and IMPUTE, respectively. SNPs are plotted according to accuracy and MAF. Black lines denote the three genotype frequencies of a SNP in Hardy-Weinberg equilibrium given its MAF.

The optimal trade-off between computing time and imputation quality was obtained by using 300 trees and five iterations. In this setting, RF imputation required 5 minutes on a quad-core computer with a 2.33 GHz processor. The mean accuracy amounted to 62.70% with a standard deviation of 17.88%. The minimal and maximal accuracy was 34.78% and 97.32%, respectively. Imputation accuracy and MAF of a SNP were found to be strongly correlated as shown in Figure 2. Only SNPs with a small MAF showed a high imputation quality. Considering SNPs with a higher MAF, the accuracy decreased drastically. Accuracy of imputed SNPs with a MAF between 0.15 and 0.3 is heterogeneous. Given the MAF of a SNP, imputation accuracy is similar to the maximum of genotype frequencies of a SNP in Hardy-Weinberg equilibrium (Figure 2).

IMPUTE required 20 minutes computing time on a computer with a 2.33 GHz processor. The mean accuracy was 92.62%, with a standard deviation of 10.61%. The minimal and maximal accuracy was 52.49% and 100.00%, respectively. MAF and accuracy were not found to be correlated (Figure 2).

## Discussion and conclusion

The RF imputation procedure consumes an acceptable amount of computing time and imputes considerably faster than the alternative standard approach. An imputation of a full GWAS SNP data set might be feasible even on slow computers.

However, this advantage is accompanied by a lower quality of imputation compared to IMPUTE. Obviously, the imputation quality of a SNP strongly depends on its MAF. Further theoretical study is needed to investigate whether the expected imputation accuracy of a SNP can be roughly estimated by calculating the maximum of its genotype frequencies.

To conclude, we presented an approach of imputing untyped SNPs using RF. The procedure is computationally feasible. However, for a highly accurate imputation of untyped SNPs, alternative methods may be more appropriate.

## List of abbreviations used

GAW: Genetic Analysis Workshop; GWAS: Genome-wide association study; LD: Linkage disequilibrium; MAF: Minor allele frequency; RF: Random forests; SNP: Single-nucleotide polymorphisms.

## Competing interests

The authors declare that they have no competing interests.

## Authors' contributions

DFS carried out the analysis, the programming of all C++/C/R program code, and drafted the manuscript. SS merged HapMap data and Framingham Heart Study data and drafted the manuscript. IRK participated in the design and coordination of the study. AZ conceived of the study and finalized the manuscript.
